# Comparison of the Virulence of Korean African Swine Fever Isolates from Pig Farms during 2019–2021

**DOI:** 10.3390/v14112512

**Published:** 2022-11-13

**Authors:** Ki-Hyun Cho, Seong-Keun Hong, Min-Kyung Jang, Ji-Hyoung Ryu, Hyun-Jeong Kim, Yu-Ran Lee, In-Soon Roh, Hyun-Joo Sohn, Hae-Eun Kang, Jee-Yong Park

**Affiliations:** Foreign Animal Disease Division, Animal and Plant Quarantine Agency, 177 Hyeoksin 8-ro, Gimcheon 39660, Korea

**Keywords:** African swine fever, animal experiment, virulence, South Korea

## Abstract

African swine fever (ASF) was first reported in South Korea in September 2019, and as of 31 December 2021, a total of 21 cases in domestic pig farms and 1875 ASFV-infected wild boars have been confirmed in the country. With the continued circulation of ASF in wild boars, and subsequent outbreaks in domestic pigs, concerns were raised about the possible changes in virulence occurring among African swine fever viruses (ASFV) circulating in South Korea. In this study, four Korean ASFV strains isolated from domestic pig farms at different time points between 2019 and 2021 were chosen, and used to experimentally infect domestic pigs by intramuscular inoculation to compare their virulence. All challenged pigs died at 4–9 days post-inoculation, with many showing clinical symptoms of fever, depression, loss of appetite, and recumbency. Gross lesions observed at necropsy included enlargement and hemorrhage of the lymph nodes and hydropericardium. The study showed that all four Korean ASFV isolates caused acute forms of illness, which supports the view that virulence among the circulating ASFV isolates in South Korea remained unchanged and highly virulent during this period.

## 1. Introduction

African swine fever (ASF) is an infectious viral disease affecting swine species. ASF is characterized by acute hemorrhagic fever and death. The etiological agent of ASF is the ASF virus (ASFV), which belongs to the family *Asfarviridae*, genus *Asfivirus.* ASF was first reported in Kenya by Montgomery in 1921 [1], and has historically been endemic to sub-Saharan African countries. However, ASF was transmitted to Georgia in 2007 and has expanded to many countries in Europe, Asia, and the Americas. Notably, China reported the first ASF outbreak in August 2018 [2], which spread to all provinces within only 9 months, while continuing to propagate across the neighboring Asian countries, including Mongolia, Vietnam, Cambodia, North Korea, Laos, the Philippines, Myanmar, East Timor, and Indonesia (WOAH WAHIS interface, visited online on 3 June 2022).

In South Korea, 14 ASF cases in domestic pig farms, primarily in the northwestern border regions with North Korea, were reported from 16 September 2019 to 9 October 2019 [3]. On 3 October 2019, an ASFV-infected wild boar was also found in the demilitarized zone in the northwestern border region [4]. Despite government efforts to contain ASF spread in wild boars, regions with infected wild boars have continued to expand eastward and southward along the mountain ranges. As of 31 December 2021, a total of 1875 infected wild boars have been confirmed across Gyeonggi, Gangwon and Chungcheongbuk Provinces. Of concern was the southward spread to two counties in Chungcheongbuk Province, where 53 ASFV-infected wild boars were found from November to December 2021. As ASF continued its expansion in wild boars, sporadic outbreaks in domestic pig farms continued to be reported in regions where infected wild boars have been detected. In 2020, two ASF cases in pig farms were detected in Gangwon Province in October [5], while in 2021, a total of five outbreaks occurred in May, August, and October across Gangwon Province.

With the continued circulation of ASFV in the wild boar population in South Korea, there is a need to monitor the changes in genetic characteristics and virulence that may occur over time. Changes in virulence have previously been reported in several countries in Eastern Europe, such as Latvia and Estonia, where ASFV isolated from wild boars showed reduced virulence [6,7,8]. Many of the attenuated ASFV strains are non-hemadsorbing (HAD) phenotypes due to mutations or deletions in the EP402R gene encoding CD2V protein.

In South Korea, ASFVs with variations in the intergenic region (IGR) between I73R and I329L have been reported in wild boars [4], and although such findings have raised concerns regarding the possible emergence and circulation of ASFV with reduced virulence, information on the genetic characteristics and virulence of ASFV in South Korea, in particular for wild boars, is limited.

Understanding the virulence of ASFV isolates circulating in South Korea is essential for establishing and maintaining effective control measures. In particular, ASF surveillance strategies for many countries, including South Korea, are more focused on quickly identifying ASF with acute forms of illness, with emphasis on clinical surveillance that encourages early notification and diagnosis, primarily by antigen detection. Major changes in the virulence of circulating ASFV isolates would require modifications to the existing control strategies. For this purpose, animal experiments were conducted to compare the virulence of four ASFV isolates from domestic pig farms between 2019 and 2021, including the clinical signs, disease dynamics, and post-mortem lesions in the infected pigs. Considering that the outbreaks in domestic pigs are likely to be spillover infection from the ASFV isolates circulating in wild boars, this information should also provide an insight into the virulence of circulating viruses in wild boars.

## 2. Materials and Methods 

### 2.1. Virus

In South Korea, many of the infection locations had a pig or pigs with clinical signs of fever, loss of appetite, depression, nasal hemorrhage, abortion, or death. Major gross lesions at necropsy included infarction and enlargement of the spleen, enlargement and hemorrhage in lymph nodes, and petechia in kidneys. Genetic characterization showed that the 16 ASFVs isolated during 2019–2020 belonged to the p72 genotype II and serogroup 8. There were no mutations or deletions in the EP402R genes of 16 isolates [5]. The other 5 isolates from pig farms during 2021 were also classified as the p72 genotype II (accession numbers OP795747, OP795748, OP795749, OP795750, OP795751) and serogroup 8 (accession numbers OP795752, OP795753, OP795754, OP795755, OP795756).

Four ASFV isolates were selected for the virulence comparison study. Isolates from the first notified ASF outbreak farm from each of the years 2019, 2020, and 2021 were considered to be representative of their respective years, as they are considered to be new spillover infections from ASFVs circulating in wild boars. Furthermore, they represented the widest time interval between outbreaks, with the earliest outbreak in a domestic pig farm being reported in October and the next outbreak in either May or October of the following year. The 3 isolates were designated as Korea/Pig/Paju1/2019, Korea/Pig/Hwacheon1/2020, and Korea/Pig/Yeongwol/2021, respectively. In addition, the isolate from the last reported ASFV-infected farm in October 2021 (Korea/Pig/Inje2/2021) was chosen for this study, representing the most recent isolate. 

All four strains were isolated from the spleens of dead pigs from each infected farm. Virus isolation was conducted according to the procedure proposed by the Center for Animal Health Research, European Union Reference Laboratory of ASF. In short, spleen added with phosphate buffer saline and gentamicin was homogenized and centrifuged, after which supernatant was filtered for virus isolation in porcine alveolar macrophages (PAMs). PAMs were obtained via bronco–alveolar lavage of the lungs of 2–3-week-old piglets. PAMs were seeded into a 96-well tissue plate with RPMI 1640 and 10% fetal bovine serum for 4 h at 37 °C in a CO_2_ incubator. Prepared spleen homogenate was incubated in PAMs for 1 h at 37 °C in a CO_2_ incubator and discarded. RPMI 1640 media and 1% swine erythrocyte suspension were added. The plate was incubated at 37 °C in a CO_2_ incubator and the potential presence of HAD was observed for 6 days. All 21 ASFV strains clearly showed HAD. The inoculated virus was prepared through two passages in PAMs. Titrations of inoculum were performed using a hemadsorption assay to monitor the end point of dilution of the ASFV isolate in PAMs. Briefly, 1 × 10^5^ PAMs were seeded into each well of a 96-well plate with an addition of RPMI 1640 medium with 10% fetal bovine serum and fifty microliters of 10-fold serial dilutions of the inoculum were added into each well, followed by the addition of 1% swine red blood cells. After 6 days of incubation at 37 °C in a CO_2_ incubator, 50% hemadsorbing doses (HAD_50_) were estimated according to the method of Reed and Muench.

### 2.2. Animal Experiment

Twelve 8-week-old landrace pigs from a commercial pig farm, which was negative for ASFV antigens and antibodies, were used in this study. The farm of origin was a farrow-to-finish farm that was free of ASF, foot–and–mouth disease, classical swine fever, porcine respiratory and reproductive syndrome, and porcine epidemic diarrhea, and where routine vaccination programs for porcine circovirus 2, classical swine fever virus, foot-and-mouth disease virus, and *Mycoplasma hyopneumoniae* was conducted. Animal experiments in this study were conducted in the Animal Biosafety Level 3 (ABSL3) of Animal and Plant Quarantine Agency (APQA), Gimcheon. This experiment was approved by the Animal Ethics Committee of APQA (authorization no. 2021-635) to ensure the ethical and humane treatment of experimental animals. 

To compare the virulence of the four isolates, four groups consisting of three pigs were inoculated by intramuscular (IM) injection with a 10^3^ HAD_50_ dose of their respective strains. Groups 1, 2, 3, and 4 were inoculated with Korea/Pig/Paju1/2019, Korea/Pig/Hwacheon1/2020, Korea/Pig/Yeongwol/2021, and Korea/Pig/Inje2/2021, respectively.

A humane endpoint was determined when pigs displayed severe clinical signs of fever, anorexia, recumbency, respiratory difficulty, and vomiting for more than two consecutive days, or when the clinical scoring system used in this study exceeded 18 points, as was applied in a previous study [8]. The pigs were euthanized by IM injection of Zoletil^®^ and Rompun^®^ for deep anesthesia and exsanguination. Dead pigs were immediately subjected to necropsy.

### 2.3. Clinical Evaluation and Sample Collection

All challenged pigs were examined daily until their death. Clinical evaluation was based on the previously described clinical score guidelines for ASF [9]. In brief, the scoring is based on ten different categories, including rectal temperature, loss of appetite, recumbency, skin hemorrhage, joint swelling, labored breathing and/or cough, ocular discharge, diarrhea, hematuria, and vomiting. The maximum score is 40 points. 

Samples, including whole blood with EDTA, serum, and swabs from the oral and nasal cavity and rectum, were collected from the day of inoculation to the day before death. Pigs were manually restrained for sample collection. Whole blood and serum samples were collected from the jugular vein. Swab specimens were collected using sterile cotton swabs in a transport medium (Noble Biosciences, Hwaseong, South Korea). All pigs were subjected to necropsy soon after euthanasia or death. Tissue collected during necropsy included spleen, liver, kidney, heart, forelimb muscle, and the submaxillary, inguinal, mesenteric, hilar, and gastrohepatic lymph nodes.

### 2.4. ASFV Genome Detection

Whole blood, tissues, and oral, nasal, and rectal swabs from infected pigs were screened for ASFV using OIE TaqMan quantitative polymerase chain reaction (qPCR) [10]. DNA was extracted using Maxwell^®^ RSC whole blood DNA, and Maxwell^®^ RSC viral total nucleic acid purification kit (Promega, Madison, WI, USA), for whole blood and tissue homogenate and swab samples, respectively. The extraction was conducted using a Maxwell^®^ RSC 48 instrument according to the manufacturer’s instructions. OIE TaqMan qPCR was performed using Bio-Rad CFX-96 (Bio-Rad Laboratories, Hercules, CA, USA). Samples with a recorded cycle threshold (Ct) < 40.0 were considered positive, while samples with no recorded Ct value were considered negative. Viral genome load was estimated by correlation with the Ct values using OIE TaqMan qPCR and copy numbers of the synthetic positive control of pUC57 vector inserted with 378 bp of B646L gene of BA71V strain.

### 2.5. Detection of Antibodies against ASFV

All sera were screened for the presence of ASFV antibodies with a commercial ELISA kit, Eurofins Ingenasa^®^-INGEZIM PPA COMPAC K3 kit (Eurofins Ingenasa, Madrid, Spain), according to the manufacturer’s instructions. Samples with positive or inconclusive results by the ELISA screening test were confirmed by the immunoperoxidase test (IPT) according to the standard protocols provided by the European Reference Laboratory for ASF.

## 3. Results

### 3.1. Clinical Signs Observed, and Survival

**Figure 1:**
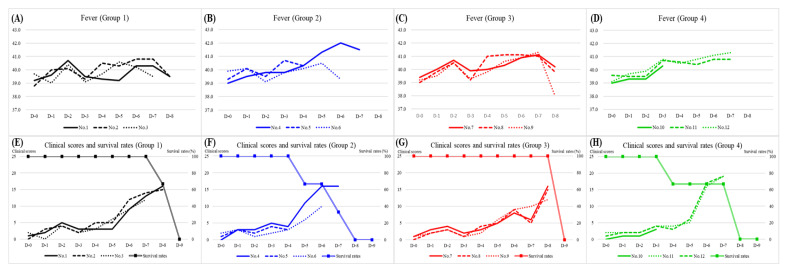
Results of rectal temperature, clinical score, and survival rate in four groups. Change of rectal temperature (**A**–**D**) and clinical scores and survival rates (**E**–**H**) of four groups.

In Group 1, fever (>40 °C) was first detected at 5.0 ± 1.0 (4–6) days post-inoculation (DPI). Clinical signs were observed at 5.0 ± 1.0 (4–6) DPI (Table 1). Inoculated pigs commonly displayed fever, depression, loss of appetite, and recumbency. Pig no. 1 showed respiratory distress, diarrhea, and ocular discharge (Table 2). The clinical score increased from 12 to 16 points immediately before death. The temperature was below 40 °C in all three pigs one day prior to their death (Figure 1A). All pigs in the group died at 8.7 ± 0.6 (8–9) DPI (Table 1).

The disease dynamics of groups 2, 3, and 4 were similar to those of group 1. In the three groups, the rectal temperature increased to above 40 °C at 3–6 DPI (Figure 1B–D). Clinical manifestations were observed from 4 to 5 DPI (Table 1). The common clinical signs were fever, depression, inappetence, and recumbency, except for pig no. 5 in group 2 and pig no. 10 in group 4, which died without clear clinical signs at 5 and 4 DPI, respectively. Both pigs showed fever (>40 °C) from 3 DPI to death. Clinical symptoms such as ocular discharge, diarrhea, breathing difficulty, and melena were observed in some pigs (Table 2). The clinical scores of all pigs, excluding no. 5 and no. 10, continued to increase to more than 10 points before death. All pigs died within 9 (4–9) DPI (Table 1E–1H).

**Figure 2:**
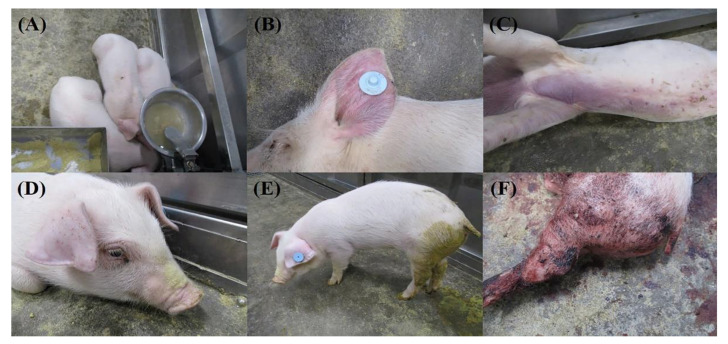
Clinical signs of pigs infected with four ASFV isolates from South Korea from 2019 to 2021. (**A**) Group 1 pigs huddling together at 5 DPI, (**B**) skin hemorrhage on the left ear of pig no. 4 at 5 DPI, (**C**) skin hemorrhage on the trunk of pig no. 7 at 4 DPI, (**D**) ocular discharge in pig no. 4 at 5 DPI, (**E**) diarrhea in pig no. 4 at 4 DPI, (**F**) bloody diarrhea in pig no. 5 at 4 DPI.

Clinical characteristics and signs of each pig are summarized in Table 1 and Table 2. Rectal temperature, clinical score, and survival rate are shown in Figure 1. Clinical signs that were frequently observed are shown in Figure 2.

### 3.2. Viral Loads in Blood, Virus Shedding via Oral, Nasal, and Rectal Routes, and Antibody Detection

**Figure 3:**
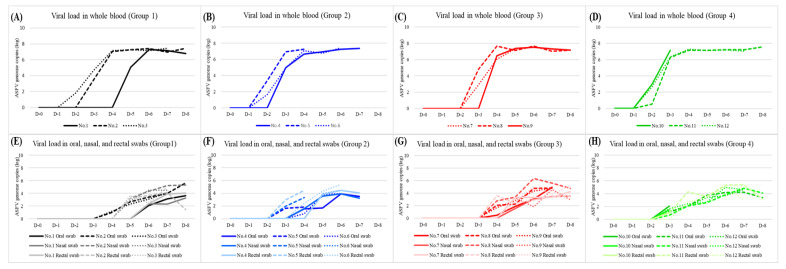
Detection of ASFV genome in whole blood and oral, nasal, and rectal swab samples from the four groups. Viral loads in whole blood (**A**–**D**) and in oral, nasal, and rectal swabs (**E**–**H**) of four group.

In group 1, the ASFV genome was detected in blood at 3.3 ± 1.5 (2–5) DPI, which was 1–3 days earlier than the onset of fever. The viral loads were 10^1^–10^5^ copies on the first day of detection and increased to approximately 10^8^ copies within 1–2 days. These high levels were maintained until death (Figure 3A). Pigs in group 1 began excretion of the virus via oral and nasal routes at 4.7 ± 1.2 (4–6) and 5.3 ± 0.6 (5–6) DPI, respectively, which was 1–3 days after the detection of viremia. Viral loads of 10^1^–10^3^ copies in oral and nasal swabs were observed on the day of detection, and their levels reached more than 10^4^ copies. Rectal shedding started at 5.3 ± 0.6 (5–6) DPI. Its maximum viral load was 10^4^–10^5^ copies. ASFV genome in the blood, and oral, nasal, and rectal swabs, was detected continuously from their onset to death (Figure 3E).

Groups 2, 3, and 4 also showed similar patterns of viremia and viral shedding via oral, nasal, and rectal routes. Viral loads in the blood were detected at 2–5 DPI in these groups. The viral titers increased to more than 10^7^ copies within 2 days after the beginning of viremia. This high level of viremia was maintained until death (Figure 3B–D). Virus shedding via the oral route started at 3–4 DPI at a level of fewer than 10^2^ copies, which increased to 10^3^–10^4^ copies within 2 days. The maximum load in the oral swabs was 10^5^ copies. ASFV genome was detected in nasal and rectal swabs 1 day after the onset of viremia and was maintained at 10^4^–10^5^ copies until death (Figure 3F–H). Viral loads in nasal swabs from coughing pigs (Pig No. 1, 6, 7, 8, 9, and 11) and rectal swabs from pigs with bloody diarrhea (Pig No. 6, 11, and 12) are slightly higher than those from pigs without such clinical signs (Figure 3E–H). Pigs no. 5 and 10 had viremia and shed via oral, nasal, and rectal routes from 3 DPI to death. In all pigs, the ASFV antibody was negative until death. Viremia and virus shedding in the four groups are shown in Table 1 and Figure 3.

### 3.3. Viral Loads in Tissues and Post-Mortem Lesions

Seven organs, consisting of the spleen, submaxillary lymph nodes, liver, kidney, heart, lung, and forelimb muscles of all pigs were subjected to OIE TaqMan qPCR. In all groups, the viral load in the spleen was the highest at 10^6^–10^7^ copies, followed by either the lymph node or liver (10^5^–10^6^ copies), and either the kidney or heart (10^4^–10^5^ copies). Forelimb muscles had the lowest titer with 10^3^ copies. The distribution of the virus by tissue was slightly different between groups and individuals (data not shown).

**Figure 4:**
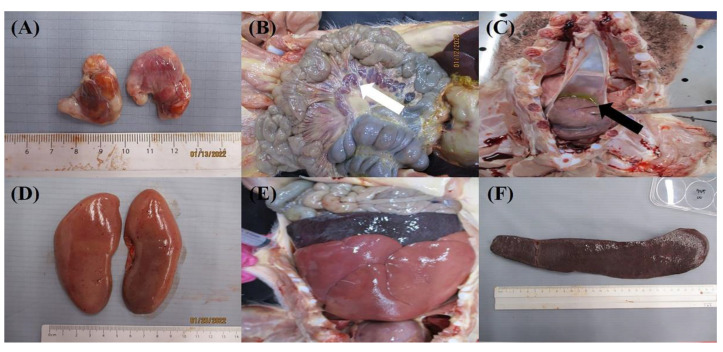
Gross lesions at necropsy. (**A**) Enlargement and hemorrhage of submaxillary lymph nodes in pig no. 1, (**B**) enlargement and hemorrhage of mesenteric lymph nodes in pig no. 3 (white arrow), (**C**) hydropericardium in pig no. 10 (black arrow), (**D**) petechiae in renal cortex in pig no. 4, and (**E**,**F**) thickened and enlarged spleen in pig no. 4.

Gross lesions commonly observed in the infected pigs included enlargement and hemorrhage of the lymph nodes and hydropericardium. All pigs, excluding pig no. 2, had petechiae in the renal cortex. Hemorrhage in the urinary bladder was observed in 10 of 12 pigs. Pathological lesions in the spleen were varied, which included thickened spleens being observed in most pigs (7/12), and enlargement (5/12), dark coloration (3/12) and friable spleen (1/12). Hemorrhage in the pericardium was observed in 7 of 12 pigs. Pathological lesions observed in some pigs were hemorrhage in the pericardium (7/12), petechiae in the stomach (6/12), ulcers in the stomach (2/12), and interstitial pneumonia (2/12). Gross lesions for each pig are summarized in Table 2. Common gross lesions observed in challenged animals are shown in Figure 4.

## 4. Discussion

Based on criteria of clinical courses and virulence of ASFV in pigs [11,12,13], all four Korean ASFV isolates were highly virulent strains causing acute form, of which mortality rate, clinical signs, survival period, and gross lesions at necropsy were similar to those of other highly virulent ASFV strains such as Georgia 2007, Armenia 07, Pig/Heilongjiang/2018, Malawi Lil-1 [2,14,15,16].

The clinical signs commonly observed in all the groups were fever, loss of appetite, depression, recumbency, and death. Skin erythema, labored breathing, ocular discharge, diarrhea, and bloody diarrhea were observed in some pigs. These signs are not specific to ASF and it is unlikely that farmers will notify the authorities based on the clinical signs alone. However, another common feature of the results was the death of all infected pigs, including two pigs (no. 5 and 10) that died suddenly without exhibiting any clear clinical signs, but displayed fever, viremia, and virus shedding via oral, nasal, and rectal routes from 3 DPI to death, indicative of ASF infection. In South Korea, outbreak farms with death in sows were more likely to be detected earlier as even one or two deaths were considered to be significant enough to warrant notification by farmers. Conversely, in grow-finisher pigs, farmers were likely to take actions after the disease had progressed further, when more significant numbers of dead pigs had been observed. A current sampling scheme for active surveillance targets the sampling of pigs with suspect clinical signs, but, as this study has shown, dead pigs remain a major characteristic of the ASF infection in South Korea. An enhanced targeted surveillance of the regular testing of select dead pigs, especially for farms located in regions with ASFV-infected wild boars, could be considered as an effective addition to improve the current overall surveillance program in South Korea.

The pathological lesions commonly observed for all pigs in the groups were enlargement and hemorrhage of the lymph nodes, and hydropericardium, which are not specific to ASF. Alternatively, markedly enlarged splenomegaly with dark color and friableness, although is a featured lesion of ASF, was not observed in all of the infected pigs. In South Korea, dead pigs in ASF-suspect farms are necropsied and lesions are examined. However, there may be a tendency by the examiner to rule out ASF in the absence of clear lesions of the spleen. Therefore, as this study has shown, despite the highly virulent nature of the ASFV isolates, pathological lesions may vary among animals, including lesions of the spleen. Pathological examination should consider all aspects of the disease and laboratory diagnosis should be conducted, even if clear lesions are not observed in the spleen.

The disease dynamics of the four Korean isolates were similar, with onset of viremia at 2–5 DPI, followed by observation of clinical signs and virus shedding via the oral, nasal, and rectal routes. All samples, including whole blood, and oral, nasal, and rectal swabs, were continuously tested positive from their first detection as was shown in other previous studies [17]. Our study showed antigen testing of EDTA whole blood to be the optimal sample for early detection of ASFV. Oral, nasal, and rectal swabs may also be an alternative sample for the diagnosis of ASF, although there was a 1–2 day delay in detection compared with whole blood samples. This corresponds to the results of previous studies [18]. In all dead pigs, ASFV genomes were detected in all organs. EDTA whole blood for diseased pigs and spleen for dead pigs should be prioritized for sampling during surveillance.

## 5. Conclusions

With the continued circulation of ASFV in wild boars in South Korea, there is a need to continuously monitor and detect changes in virulence and ascertain whether changes need to be applied to existing control measures, including surveillance.

This study of the four ASFV isolates from domestic pigs from 2019 to 2021 showed similar virulence in experimentally infected pigs. Although the ASFV isolates used in the animal experiments were isolated from domestic pigs, when considering that the outbreaks in domestic farms are likely to be spillover infection from the ASFV isolates circulating in wild boars, the study supports the view that the ASFV isolates circulating in South Korea are and remain highly virulent. Therefore, the current control measures are likely to remain effective.

The study has also identified features that should be considered to improve the current surveillance scheme, including targeted surveillance of dead pigs, training of veterinary officers performing necropsy, and selection of optimal samples.

It should be noted that in addition to the ASFV isolates circulating in South Korea, new ASFV introductions from abroad remain a major threat. This is made further complicated as the ASF situation in neighboring countries is ever-changing. In China, genotype II ASFV isolates that genetically vary from the first Chinese isolate (Pig/Heilongjiang/2018) were found during surveillance of domestic pig farms in northeastern provinces from June to December 2020. These variant strains displayed low virulence and high transmissibility, causing chronic and persistent infections [19]. Moreover, low virulent genotype I ASFV isolates were detected in fattening farms in Shandong and Henan provinces in July 2021 [20]. Introduction of such ASFV with low virulence into South Korea could lead to major changes in the ASFV isolates circulating in the county. Continuous efforts to elucidate genetic characteristics, including full genetic sequencing, and to assess the virulence of ASFV strains will be required to quickly identify such changes and contribute to the implementation of effective control policies.

## Figures and Tables

**Table 1:** Clinical characteristics and virological parameters of pigs infected with four ASFV isolates from domestic pig farms in South Korea from 2019 to 2020. All data are shown as average ± standard deviation.

Group	Inoculated ASFV Strain (Date of Outbreak)	Survival Period	Days to Onset of Fever	Clinical Signs		Viremia		Virus Shedding					
								Oral Shedding		Nasal Shedding		Rectal Shedding	
				Days to Onset	Max Score ^3^	Days to Onset	Max Titer ^3^	Days to Onset	Max Titer ^3^	Days to Onset	Max Titer ^3^	Days to Onset	Max Titer ^3^
1	Korea/Pig/Paju1/2019 (16 September 2019)	8.7 ± 0.6	5.0 ± 1.0	5.0 ± 1.0	14.3 ± 2.1	3.3 ± 1.5	7.4 ± 0.1	4.7 ± 1.2	4.5 ± 1.0	5.3 ± 0.6	4.4 ± 1.0	5.3 ± 0.6	4.4 ± 0.5
2	Korea/Pig/Hwcheon1/2020 (8 October 2020)	6.7 ± 1.5	3.7 ± 0.6	4.0 ^1^	13.0 ^1^	2.3 ± 0.6	7.4 ± 0.1	3.7 ± 0.6	3.2 ± 1.2	4.0 ± 1.0	3.9 ± 0.6	4.0 ± 1.0	4.8 ± 0.6
3	Korea/Pig/Yeongwol/2021 (5 May 2021)	9.0 ± 0.0	4.3 ± 0.6	5.3 ± 1.5	14.3 ± 2.1	3.3 ± 0.6	7.6 ± 0.1	4.0 ± 0	4.8 ± 0.1	4.7 ± 0.6	4.8 ± 1.4	4.7 ± 0.6	4.3 ± 0.8
4	Korea/Pig/Inje2/2021 (8 October 2021)	7.0 ± 2.7	3.0 ± 0	5.0 ^2^	14.5 ^2^	2.0 ± 0	7.3 ± 0.2	3.0 ± 0	3.7 ± 1.4	3.0 ± 0	3.8 ± 2.0	3.3 ± 0.6	3.9 ± 2.4

^1^ Pig no. 5, which died at 5 DPI without any clinical signs, was excluded. ^2^ Pig no. 10, which died at 4 DPI without any clinical signs, was excluded. ^3^ log_10_ genome copies.

**Table 2:** Summary of clinical signs and gross lesions in pigs inoculated with four Korean ASFV isolates.

Clinical Signs and Gross Lesions			Group 1			Group 2			Group 3			Group 4			Total Frequency	
			1	2	3	4	5	6	7	8	9	10	11	12		
Clinical signs	Survival period		9	9	8	8	5	7	9	9	9	4	9	8	-	
	Fever (>40.0 °C)		+	+	+	+	+	+	+	+	+	+	+	+	12/12	(100%)
	Loss of appetite		+	+	+	+	+	+	+	+	+	+	+	+	12/12	(100%)
	Depression		+	+	+	+	+	+	+	+	+	+	+	+	12/12	(100%)
	Skin hemorrhage		-	-	+	+	-	-	+	-	-	+	-	+	5/12	(41.7%)
	Labored breathing and/or cough		+	-	-	-	-	+	+	+	+	-	+	-	6/12	(50.0%)
	Ocular discharge		-	-	-	+	-	-	+	+	+	-	-	-	4/12	(66.7%)
	Diarrhea		+	-	-	+	-	-	+	+	-	-	-	-	4/12	(66.7%)
	Bloody diarrhea		-	-	-	-	-	+	-	-	-	-	+	+	3/12	(25.0%)
Gross lesions	Lymph nodes	Enlargement	+	+	+	+	+	+	+	+	+	+	+	+	12/12	(100%)
		Hemorrhage	+	+	+	+	+	+	+	+	+	+	+	+	12/12	(100%)
	Heart	Epicardial hemorrhage	+	-	-	+	-	+	+	+	+	-	+	-	7/12	(58.3%)
		Hydropericardium	+	+	+	+	+	+	+	+	+	+	+	+	12/12	(100%)
	Lung	Interstitial pneumonia	-	-	-	-	+	-	+	-	-	-	-	-	2/12	(16.7%)
		Interlobular edema	+	+	-	-	-	-	-	-	-	-	-	-	2/12	(16.7%)
	Thorax	Hydrothorax	-	-	-	-	-	-	-	+	-	-	-	-	1/12	(8.3%)
	Stomach	Petechiae	+	-	-	+	-	+	+	-	-	-	+	+	6/12	(50.0%)
		Ulcer	-	+	-	-	-	-	-	-	-	-	-	+	2/12	(16.7%)
	Spleen	Thickened	+	-	-	+	-	+	+	-	+	-	+	+	7/12	(58.3%)
		Dark	-	-	-	+	+	-	-	-	-	+	-	-	3/12	(25.0%)
		Enlarged	-	+	-	+	+	-	-	-	-	+	-	+	5/12	(41.7%)
		Friable	-	+	-	-	-	-	-	-	-	-	-	-	1/12	(8.3%)
	Kidney	Petechiae	+	-	+	+	+	+	+	+	+	+	+	+	11/12	(91.7%)
	Urinary bladder	Hemorrhage	+	-	+	+	-	+	+	+	+	-	+	+	9/12	(75.0%)

## Data Availability

The data presented in this study are available on request from the corresponding author. The original contributions generated for the study are included in the article. Further inquiries can be directed to the corresponding author.
